# Highlight: Unlocking the Ancient Origins of Cell Death

**DOI:** 10.1093/gbe/evad172

**Published:** 2023-10-11

**Authors:** Casey McGrath

Apoptosis, often referred to as programed cell death, is a fundamental process crucial to the growth and development of multicellular organisms. This process, or a primordial form of it, is also observed in single-celled eukaryotes like yeast and other microeukaryotes (aka protists). The origin of eukaryotic apoptosis remains an open question in biology. However, studies have noted that many apoptosis-initiating factors have a bacterial or mitochondrial origin, providing a clue into the evolutionary history of this widespread phenomenon. In a new study published in *Genome Biology and Evolution*, scientists from the Institute of Biochemistry and Biophysics of the Polish Academy of Sciences reveal that many apoptotic factors may trace their origins to the time of mitochondrial domestication, suggesting remarkable conservation over the span of 1.8 billion years (Kaushal et al. 2023).

The processes triggering apoptosis exhibit striking similarities among various diverse eukaryotes: an increase in mitochondrial membrane permeability sets in motion a cascade of events involving proteins called apoptosis-inducing factors (AIFs), kickstarting the pathway that culminates in cell death. According to phylogenetic analyses, these AIFs usually have a bacterial/mitochondrial origin. To shed further light on the evolution of apoptosis across eukaryotes, a team led by Szymon Kaczanowski and Urszula Zielenkiewicz investigated the functional conservation of apoptotic factors through a yeast complementation test. The researchers replaced each of four apoptotic genes in yeast with related proteins from diverse eukaryotes and prokaryotes. They then treated the new yeast strains with apoptosis-inducing agents to evaluate whether the introduced genes maintained the ability to induce apoptosis in yeast.

Remarkably, the study found that distantly related proteins from plants, animals, slime molds, and bacteria were largely able to functionally substitute for the original yeast proteins. “This surprising finding suggests that ancient mechanisms of cell death have been evolutionarily conserved since the domestication of mitochondria,” says Kaczanowski and Zielenkiewicz, an event that occurred approximately 1.8 billion years ago.

The study's findings further support an endosymbiotic origin of apoptosis, a hypothesis that was first proposed by Guido Kroemer in 1997 (Kroemer 1997). Kroemer suggested that the bacterial precursors of mitochondria produced both toxins (apoptotic factors) and antitoxins (anti-apoptotic factors). In this scenario, the antitoxins acted as “addiction molecules,” ensuring the persistence of the symbiont. Driven by this evolutionary conflict between bacterial endosymbionts and hosts, the toxins eventually evolved into the apoptotic factors we recognize today.

Kaczanowski and Zielenkiewicz present an alternative scenario for the evolution of apoptosis. They propose that early protoeukaryotes were predators, relying on bacterial prey. These bacteria, in response to predation, produced toxins as a defense mechanism. Over time, these bacteria were domesticated to serve as mitochondria within eukaryotic cells, and their toxins evolved into apoptotic factors. The different families of AIFs present today and their sporadic distribution across distantly related eukaryotes suggest the existence of multiple redundant toxins in the protomitochondria and hint at a coevolutionary arms race between protomitochondria and their protoeukaryotic hosts.

Regardless of whether apoptosis originates from an endosymbiotic toxin/antitoxin system or from a predator/prey dynamic, the study's findings suggest that the intricate balance between life and death within eukaryotic cells is deeply rooted in the origin of mitochondria, opening up new avenues for research into the coevolution of mitochondria and eukaryotes, as well as the ancient origins of cell death mechanisms. Furthermore, a similar approach could be used to look at other ancient cellular mechanisms beyond programed cell death and to ask to what extent conflicts among partners/participants have driven the evolution of genome features. “Future studies may reveal the evolutionary history of other aging mechanisms and could make a significant contribution to aging studies,” note Kaczanowski and Zielenkiewicz, emphasizing the broader implications of their research.
